# Climate change and the potential global distribution of *Agrobacterium tumefaciens*: a MaxEnt modeling approach for current and future climate scenarios

**DOI:** 10.3389/fpls.2026.1941149

**Published:** 2026-09-25

**Authors:** Jawaher Ibrahim Alahadeb, Mohamed S. Hodhod, Rna A. Mahran, Sameh M. H. Khalaf

**Affiliations:** 1 Department of Biology, College of Science (Majmaah), Majmaah University, Al Majmaah, Saudi Arabia; 2 Faculty of Biotechnology, October University for Modern Sciences & Arts (MSA University), October, Egypt

**Keywords:** *Agrobacterium tumefaciens*, BCC-CSM1-1, bioclimatic variables, climate change, crown gall disease, DIVA-GIS, limiting factors, MaxEnt

## Abstract

Climate change is fundamentally altering the geographic ranges of plant-pathogenic microorganisms, with profound implications for global food security and agricultural sustainability. *Agrobacterium tumefaciens*, the gram-negative soil bacterium responsible for crown gall disease in over 140 genera of dicotyledonous plants, is among the most economically significant phytopathogens worldwide, yet its macroecological distribution under current and projected future climates has never been rigorously characterised. Here, we apply the Maximum Entropy algorithm (MaxEnt) to 415 quality-filtered georeferenced occurrence records — derived from the Global Biodiversity Information Facility (GBIF) — combined with five non-collinear WorldClim 2.1 bioclimatic variables selected through Pearson correlation analysis in JMP Pro 16, to model the current potential global distribution of *A. tumefaciens* and project its future habitat suitability for 2050 and 2070 under RCP 2.6 and RCP 8.5 emission scenarios using the BCC-CSM1–1 general circulation model. The model demonstrated excellent predictive performance (mean AUC = 0.959; TSS = 0.77). Under current climate, high and very high suitability was concentrated in western and central Europe, the eastern United States, northern India, and eastern China. Jackknife analysis identified mean annual temperature (BIO1) as the dominant climatic driver, followed by precipitation of the driest month (BIO14), precipitation seasonality (BIO15), isothermality (BIO3), and mean diurnal temperature range (BIO2). Future projections consistently indicated a poleward displacement of suitable habitat, with net gains at higher latitudes and progressive contraction of very high suitability zones in current core regions, effects that intensify markedly under the high-emission RCP 8.5 scenario by 2070. Limiting factor analysis in DIVA-GIS revealed that thermal insufficiency constrains distribution at high latitudes while precipitation seasonality and dry-season moisture deficits dominate in tropical and arid zones. These findings provide a spatially explicit and climatically informed evidence base for anticipating future shifts in crown gall disease risk and informing climate-adaptive phytosanitary management strategies.

## 1 Introduction

Soil-borne bacterial pathogens represent one of the most consequential threats to global agricultural productivity, with their ecological dynamics shaped profoundly by the physical and climatic conditions of their environments (Garrett et al., 2006; Fierer et al., 2009). Among these organisms, *Agrobacterium tumefaciens* (Smith and Townsend, 1907), recently reclassified as *Rhizobium radiobacter* (Young et al., 2001), stands as one of the most widely studied and agronomically significant plant pathogens known to science (Gelvin, 2003; Nester, 2015). This gram-negative, alphaproteobacterial species is the causal agent of crown gall disease, a neoplastic disorder that afflicts more than 140 genera of dicotyledonous plants worldwide, including economically critical crops such as grapevine, stone fruit trees, roses, and a broad range of horticultural species (Burr and Otten, 1999; Pulawska, 2010). The pathogen exploits wounded plant tissue as its entry point, transferring a segment of its tumour-inducing (Ti) plasmid into the host genome, thereby hijacking the plant’s cellular machinery to produce opines and facilitate uncontrolled cell proliferation (Gelvin, 2003; Nester, 2015). The resulting galls cause significant yield losses, impair vascular function, and render affected plants vulnerable to secondary infections, making *A. tumefaciens* a pathogen of sustained scientific and economic concern (Escobar and Dandekar, 2003; Tzfira and Citovsky, 2006). We retain the widely used binomial *A. tumefaciens* throughout this manuscript, consistent with its continued and predominant use in the applied plant pathology, biosecurity, and agricultural literature, while explicitly acknowledging *Rhizobium radiobacter* as the name accepted under the current bacterial nomenclatural code (Young et al., 2001).

The geographic distribution of plant pathogens is continuously reshaped by biotic interactions, land-use change, and anthropogenic climate change, with global mean temperatures projected to rise 1.5–4.5 °C by 2100 depending on emission trajectories (IPCC, 2021). These shifts in temperature regimes, combined with alterations in precipitation patterns, humidity cycles, and extreme weather events, are fundamentally transforming the ecological envelopes within which microbial pathogens can survive, reproduce, and disseminate (Bebber et al., 2013; Garrett et al., 2006). For soil-dwelling bacteria such as *A. tumefaciens*, which depend heavily on soil temperature, moisture regimes, and pH for their survival and infectivity, climate-driven environmental changes are expected to exert particularly pronounced effects on their distributional ranges and seasonal activity windows (Agrios, 2005). Understanding how climate change might expand, contract, or shift the geographic range of this pathogen is therefore a question of mounting urgency for plant pathologists, agricultural planners, and policy makers alike.

Species distribution models (SDMs) have emerged over the past two decades as indispensable tools for characterising the ecological niches of organisms and projecting their potential distributions under both contemporary and future climatic conditions (Elith and Leathwick, 2009; Peterson et al., 2011). Among the array of SDM algorithms available, Maximum Entropy modeling — widely implemented through the MaxEnt software package — has consistently demonstrated superior predictive performance, particularly in scenarios where occurrence data are available only as presence-only (presence-background) records rather than true presence–absence data (Phillips et al., 2006; Phillips and Dudik, 2008). MaxEnt operates on the principle of finding the probability distribution of maximum entropy subject to the constraint that expected values of environmental features match their empirical averages across known occurrence localities, generating robust habitat suitability maps that can be extrapolated across geographic space and projected into future time periods (Phillips and Dudik, 2008; Merow et al., 2013). The model’s flexibility, its capacity to handle complex interactions among predictor variables, and its resilience to relatively small sample sizes have made it the method of choice in numerous studies of plant pathogens, invasive species, and organisms with sparse distributional records (Shabani et al., 2016; Shcheglovitova and Anderson, 2013).

Climate scenarios used in distribution modelling studies are typically framed around Representative Concentration Pathways (RCPs), which describe plausible future trajectories of greenhouse gas concentrations in the atmosphere (van Vuuren et al., 2011). Two contrasting pathways are of particular relevance to biodiversity and pathogen distribution research: RCP 2.6, which represents an aggressive mitigation scenario in which global warming is limited to approximately 2 °C above pre-industrial levels through decisive reductions in carbon emissions; and RCP 8.5, a high-emission business-as-usual scenario in which radiative forcing reaches 8.5 W m^-2^ by 2100, leading to severe warming, sea-level rise, and widespread ecological disruption (Moss et al., 2010; Stocker et al., 2013). Comparing projected species distributions under these two scenarios provides a powerful framework for quantifying the range of plausible ecological futures and for communicating the potential consequences of climate inaction to decision-makers. Time horizons of 2050 and 2070 are conventionally used in such analyses because they are close enough to allow reasonable confidence in climate projections yet distant enough to capture the full trajectory of ecological change that will unfold under different emission pathways (Thuiller et al., 2008; Warren et al., 2018).

Despite its global agricultural significance, and to the best of our knowledge, relatively few studies have applied spatial modeling techniques to characterise the current climatic niche of *A. tumefaciens* or to project how climate change might alter its distribution across the coming decades. The available literature has largely focused on the molecular mechanisms of pathogenesis, the ecology of Ti plasmid transfer, and the development of biological control agents, leaving the macroecological dimension of this pathogen’s distribution almost entirely unexplored (Kerr, 1980; Vrain and Copeman, 1987; Sule, 1978). This gap is particularly striking given the extensive literature applying SDMs to fungal plant pathogens such as *Fusarium* spp., *Phytophthora* spp., and *Puccinia* spp., as well as bacterial pathogens including *Xanthomonas* and *Ralstonia solanacearum* (Kriticos et al., 2011; Maczey et al., 2016; Hijmans and Graham, 2006). Filling this gap requires a rigorously constructed SDM that integrates georeferenced occurrence records with high-resolution bioclimatic data and validated model outputs capable of generating reliable predictions across both current and future time horizons.

The climatic variables that govern the distribution of soil-borne bacterial pathogens are multidimensional and often interact in non-linear ways. Temperature variables — including mean annual temperature, temperature seasonality, maximum temperature of the warmest month, and minimum temperature of the coldest month are widely recognised as primary drivers of microbial biogeography because they directly influence cell membrane fluidity, enzyme kinetics, and metabolic rates (Alqahtani et al., 2025; Fierer et al., 2009). Moisture-related variables, such as annual precipitation, precipitation of the driest quarter, and potential evapotranspiration, further modulate bacterial survival in soil by determining the water potential and aeration status of the rhizosphere (Or et al., 2007; Tecon and Or, 2017). Soil pH, texture, and organic matter content have also been implicated as modulators of *A. tumefaciens* persistence and virulence, although these edaphic variables are often correlated with the climatic predictors captured by standard bioclimatic datasets such as WorldClim and CHELSA (Fick and Hijmans, 2017; Karger et al., 2017). Identifying which among these climatic variables exerts the greatest control over the current distribution of the pathogen and which will drive range shifts under warming scenarios is essential for generating ecologically interpretable model outputs that can be translated into practical management recommendations.

In light of the foregoing considerations, the present study was designed to address a clear and pressing scientific need: a rigorous, spatially explicit assessment of the climatic niche and distributional dynamics of *Agrobacterium tumefaciens* under both current and projected future climatic conditions. Specifically, this work aims to (i) model the current global distribution of *A. tumefaciens* using the MaxEnt algorithm calibrated with georeferenced occurrence records and bioclimatic predictor variables derived from the WorldClim database; (ii) project the predicted habitat suitability of this pathogen into the future time slices of 2050 and 2070 under two contrasting Representative Concentration Pathways, namely the low-emission mitigation scenario RCP 2.6 and the high-emission business-as-usual scenario RCP 8.5; and (iii) identify and rank the key climatic variables that most strongly determine the present and future distribution of the pathogen, thereby providing an ecologically grounded understanding of how thermal and moisture gradients structure its climatic niche. By delivering spatially explicit predictions of potential range expansion, contraction, or shift, this study seeks to provide plant health authorities, agricultural extension services, and crop protection researchers with an evidence base for anticipating and mitigating the future burden of crown gall disease in a changing climate.

## 2 Materials and methods

### 2.1 Occurrence data collection and filtering

Georeferenced occurrence records of *Agrobacterium tumefaciens* were obtained from the Global Biodiversity Information Facility (GBIF; https://www.gbif.org), which aggregates specimen-level observation data from herbaria, culture collections, field surveys, and published literature archives worldwide (GBIF Secretariat, 2023); the specific occurrence dataset compiled for this study follows the taxonomic query and filtering criteria described below, and a permanent GBIF download DOI documenting the exact record set will be archived and cited in the data availability statement upon acceptance, following standard GBIF citation practice. An initial query using the accepted taxonomic name and its synonyms including *Rhizobium radiobacter* — returned a broad dataset encompassing records from multiple continents. To ensure that only spatially and ecologically reliable data were retained, a rigorous multi-step filtering protocol was applied. Records lacking precise coordinate information, those with coordinate uncertainty exceeding 10 km, duplicate entries falling within the same grid cell, and occurrences flagged by GBIF automated quality controls as geographically suspect or transposed were all excluded (Robertson et al., 2016). Additionally, records assigned to greenhouse environments, laboratory culture collections, or experimental inoculation sites were removed, as they do not reflect the natural distributional range of the organism. Whereas the preceding step removed only exact duplicate records sharing a single raster grid cell, spatial thinning is a distinct procedure that enforces a minimum nearest-neighbour distance across the entire retained dataset regardless of duplication, thereby reducing spatial autocorrelation and clustering-driven sampling bias that can otherwise inflate apparent model performance and artificially elevate predicted suitability in densely sampled regions (Boria et al., 2014). This thinning was performed using a 10-km minimum inter-point distance, matching the spatial resolution of the WorldClim predictor layers used for modelling. Following this comprehensive cleaning and thinning procedure, a final dataset of 415 georeferenced occurrence records was retained. These records, distributed across diverse climatic zones in Europe, North America, South America, Asia, Africa, and Australasia, were considered sufficient to characterise the global climatic niche of the pathogen and to support robust MaxEnt modeling at the continental and global scales. The full list of retained occurrence coordinates is provided as **Supplementary Table S1** to support transparency and reproducibility, and a georeferenced map of the training localities is provided as **Supplementary Figure S1**.

### 2.2 Bioclimatic predictor variables

Climatic predictor variables were sourced from the WorldClim 2.1 database (Fick and Hijmans, 2017), which provides global climatological surfaces at a spatial resolution of 2.5 arc-minutes (~5 km at the equator) derived from weather station observations compiled over the reference period 1970–2000. The full suite of 19 bioclimatic variables (BIO1–BIO19) was initially considered as candidate predictors, covering a broad spectrum of biologically meaningful temperature and precipitation indices (Khalaf et al., 2024a, 2024b). Raster layers for each variable were prepared in ASCII (.asc) grid format using ArcGIS 10.8 (Environmental Systems Research Institute, Redlands, CA, USA) (ESRI, 2020) to ensure compatibility with the MaxEnt modeling platform. All layers were clipped to a common geographic extent and resampled to a uniform resolution to prevent artifacts arising from cell misalignment. Bathymetric and ocean cells were masked, ensuring that model calibration was confined exclusively to terrestrial environments.

### 2.3 Future climate scenarios

Projected future climate surfaces were obtained for two time periods — the 2050s (average for 2041–2060) and the 2070s (average for 2061–2080) — under two Representative Concentration Pathways: RCP 2.6, a stringent mitigation pathway, and RCP 8.5, a high-emission baseline scenario (van Vuuren et al., 2011; Moss et al., 2010). Future bioclimatic surfaces were derived from the BCC-CSM1–1 general circulation model (GCM), developed by the Beijing Climate Centre of the China Meteorological Administration (Wu et al., 2014). The BCC-CSM1–1 model was selected on the basis of its demonstrated skill in simulating large-scale temperature and precipitation patterns across both tropical and extra-tropical regions, as well as its widespread use in species distribution modeling studies employing the WorldClim framework (Taylor et al., 2012). Future climate layers were downloaded from the WorldClim portal and processed in ArcGIS 10.8 following the same preparation workflow applied to the baseline data, including format conversion to ASCII, extent clipping, and resolution harmonisation.

### 2.4 Multicollinearity assessment and variable selection

A critical preliminary step before MaxEnt modeling is the identification and elimination of highly correlated predictor variables, which can introduce redundancy into the model and compromise the ecological interpretability of model outputs (Dormann et al., 2013). Pairwise Pearson correlation coefficients were computed among all 19 bioclimatic variables at the 415 occurrence localities using JMP Pro 16 (SAS Institute Inc., Cary, NC, USA) (SAS Institute Inc, 2021). Variables exhibiting pairwise correlation coefficients with an absolute value of |r| ≥ 0.80 were considered to be collinear and sequentially removed based on lesser ecological relevance to soil bacterial distribution. This filtering process identified strong collinearity among several temperature-related variables and among precipitation variables associated with wet and dry seasons. Following this process, a final set of five bioclimatic variables BIO1 (mean annual temperature), BIO2 (mean diurnal range), BIO3 (isothermality), BIO14 (precipitation of the driest month), and BIO15 (precipitation seasonality) was retained as the predictor set for MaxEnt modeling. These variables collectively capture the thermal regime, temperature variability, and moisture seasonality patterns most relevant to the soil-dwelling ecology and physiological tolerances of *A. tumefaciens* (Alqahtani et al., 2025; Tecon and Or, 2017; Khalaf et al., 2025).

### 2.5 MaxEnt modeling

The Maximum Entropy algorithm, as implemented in MaxEnt version 3.4.4 (Phillips et al., 2006; Phillips and Dudik, 2008), was employed to model the current potential distribution of *Agrobacterium tumefaciens* and to project its future geographic range under the four climate change scenarios. The model was run with 10 replicate bootstrap runs, and model outputs were averaged across replicates to produce final suitability maps. A random 75% of occurrence records was used for model training, with the remaining 25% reserved as an independent test set. The regularization multiplier was set to its default value of 1.0, and all feature types linear, quadratic, product, threshold, and hinge were enabled. Default regularization was retained rather than performing exhaustive parameter tuning because comparative evaluations indicate that MaxEnt’s default settings perform robustly for species with broad, well-sampled distributions and yield predictions comparable to tuned models in many empirical cases (Phillips and Dudik, 2008; Merow et al., 2013). We nonetheless recognise, in line with current best-practice recommendations (Warren and Seifert, 2011; Radosavljevic and Anderson, 2014), that systematic tuning of the regularization multiplier and feature-class combination using established frameworks such as ENMeval or kuenm would further optimise the bias–variance trade-off of the model, and we identify this as a priority for future refinement (see Discussion, Limitations). Background pseudoabsence points (n = 10,000) were randomly sampled from the calibration extent (the accessible area, M), defined as a 500-km buffer around all occurrence localities. This distance was chosen to encompass the broader climatic and dispersal neighbourhood plausibly accessible to a globally distributed, human-associated soil bacterium disseminated primarily through the movement of infected plant propagation material, while excluding climatically and biogeographically unconnected regions from the background sample, consistent with recommended approaches for defining M in correlative niche models (Barve et al., 2011). Model outputs were expressed as logistic habitat suitability scores ranging from 0 to 1, and continuous suitability surfaces were subsequently converted to binary presence/absence maps using the maximum training sensitivity plus specificity (MTSS) threshold [the mean MTSS logistic threshold obtained across the 10 bootstrap replicates, to be inserted here by the authors from the original MaxEnt output (maxentResults.csv), is reported in Section 3.2]. This threshold lies close to the lower boundary of the descriptive “Low” suitability class (0.20) used for cartographic visualisation (Section 2.9), so that grid cells classified as “Unsuitable” in the continuous five-class maps correspond closely to those classified as absence in the binary MTSS maps.

### 2.6 Variable importance and response curves

To identify the relative contribution of each bioclimatic variable to model predictions, two complementary analytical approaches embedded within MaxEnt were applied. First, the percentage contribution of each variable to the regularised training gain was computed as a direct measure of variable importance during model fitting (Phillips et al., 2006). Second, a jackknife test of variable importance was performed to assess the gain achieved by models built using each variable in isolation (single-variable gain) as well as the loss in training gain observed when each variable was omitted from the full model (Beck et al., 2014; Merow et al., 2013). Response curves relating predicted habitat suitability to individual predictor values, holding all other variables at their mean, were also generated for each selected variable to characterise the shape and directionality of the modelled species–environment relationships.

### 2.7 Limiting factor analysis using DIVA-GIS

To provide a spatially explicit identification of the primary environmental constraints governing the distribution of *A. tumefaciens* at the global scale, a limiting factor analysis was conducted using DIVA-GIS version 7.5 (Alqahtani et al., 2025; Hijmans et al., 2012). The BIOCLIM envelope model implemented in DIVA-GIS was applied to the occurrence records and selected bioclimatic layers to compute the limiting factor map, which assigns to each grid cell the identity of the single bioclimatic variable that most strongly limits the suitability of that cell relative to the conditions observed across the occurrence dataset (Khalaf et al., 2026; Alkhalifah et al., 2022; Alenezi et al., 2026). This analysis produces a categorical map in which each cell is coloured according to the most restrictive environmental variable, providing an intuitive spatial visualisation of which climatic constraints are principally responsible for excluding the pathogen from currently unsuitable regions.

### 2.8 Model validation

Model predictive performance was rigorously evaluated using two complementary metrics: the Area Under the Receiver Operating Characteristic Curve (AUC) and the True Skill Statistic (TSS). The AUC measures the probability that a randomly selected presence location will be assigned a higher predicted suitability value than a randomly selected background point, with values above 0.70 considered acceptable, above 0.80 good, and above 0.90 excellent (Fielding and Bell, 1997). The TSS is calculated as sensitivity + specificity − 1 and ranges from −1 to +1, with values above 0.4 indicating good model performance and values above 0.7 indicating excellent performance; unlike AUC, TSS corrects for predictions arising by chance (Allouche et al., 2006). Both metrics were computed for each of the 10 bootstrap replicates, and mean values with standard deviations were reported to characterise model stability across replicates. TSS was calculated at the model-specific MTSS threshold for each replicate individually before averaging, consistent with the threshold-dependent nature of the statistic (Allouche et al., 2006).

### 2.9 Spatial data preparation and visualisation in ArcGIS

All spatial data preparation, format conversion, and cartographic visualisation were carried out within the ArcGIS 10.8 environment (ESRI, 2020). Raw bioclimatic raster layers were imported, resampled to a consistent spatial resolution of 2.5 arc-minutes, clipped to the global terrestrial extent, and exported in ASCII grid (.asc) format. Following model execution, the continuous habitat suitability raster outputs produced by MaxEnt were re-imported into ArcGIS for thematic mapping. Colour-classified suitability maps were produced for the current baseline period and for each of the four future scenarios using a standardised five-class graduated colour scheme ranging from unsuitable (0–0.20) to very high suitability (0.80–1.00); this descriptive scheme, used for cartographic visualisation of the continuous suitability surface, is numerically consistent with the binary MTSS threshold applied for area calculations and change-detection analysis (Sections 2.5 and 2.8), as detailed above. Binary maps delineating the boundaries of suitable habitat under each scenario were overlaid to quantify the projected net change in suitable area between current and future periods. All maps were produced using the WGS 1984 geographic coordinate system and exported at a resolution of 300 dpi for publication.

## 3 Results

### 3.1 Multicollinearity assessment and selection of bioclimatic predictors

The pairwise Pearson correlation analysis conducted in JMP Pro 16 across all 19 WorldClim bioclimatic variables at the 415 georeferenced occurrence localities of *Agrobacterium tumefaciens* confirmed extensive multicollinearity among a large proportion of the candidate predictors, particularly within the temperature (BIO1, BIO5, BIO6, BIO10, BIO11) and precipitation (BIO12, BIO13, BIO16, BIO17) variable clusters (**Figure 1**); the full matrix of pairwise correlation coefficients is reported in **Figure 1** and is not reproduced in the text. After applying the |r| ≥ 0.80 collinearity threshold and integrating biological reasoning, five non-collinear predictors were retained: BIO1, BIO2, BIO3, BIO14, and BIO15. The independent explanatory contribution of these five variables was subsequently corroborated, though not formally validated, by the jackknife test of variable importance and by the response curve analyses reported in Section 3.3; jackknife analysis evaluates the contribution of variables within the fitted model and is complementary to, rather than a substitute for, the correlation-based selection procedure used here.

**Figure 1:**
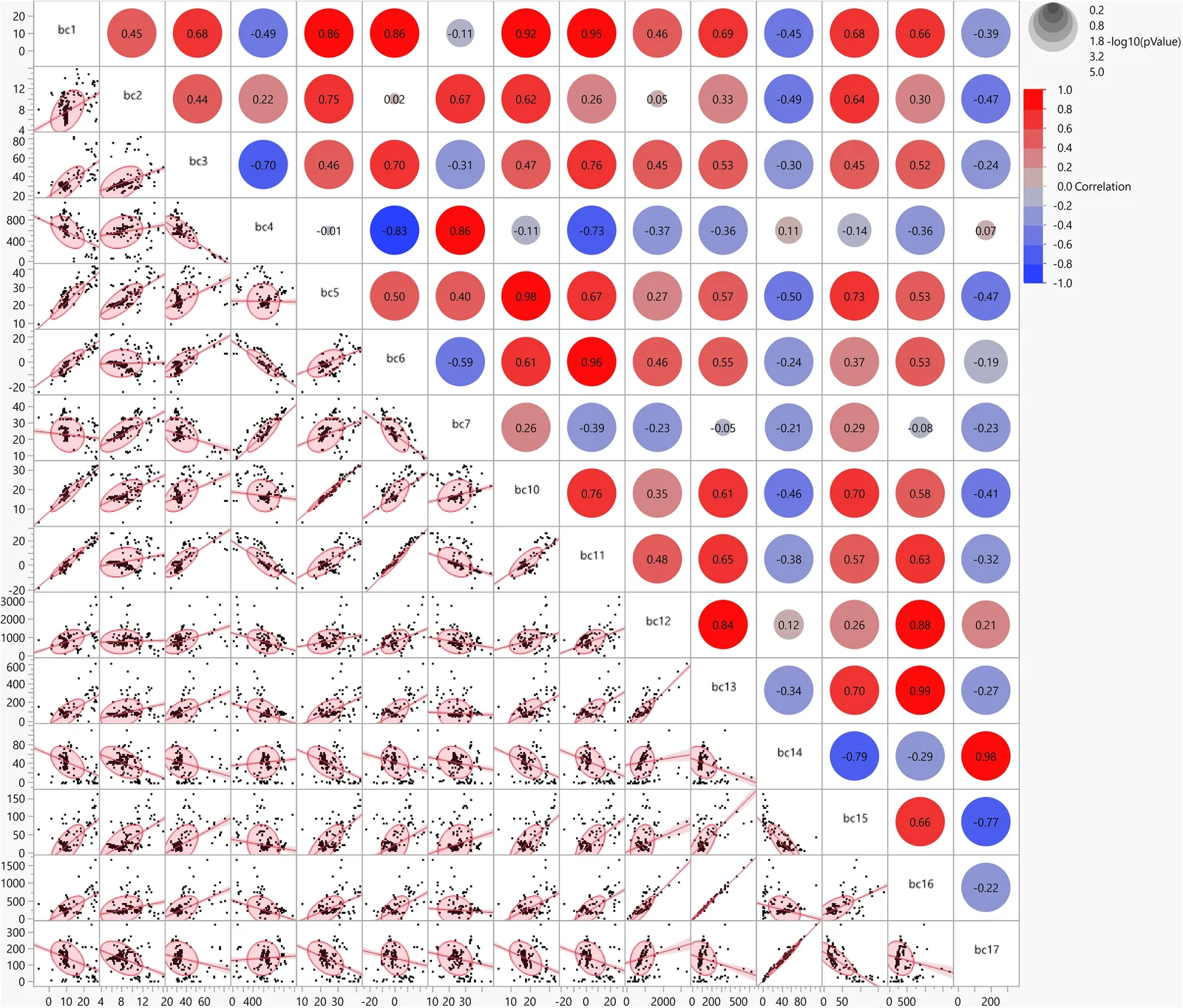
Multivariate Pearson correlation matrix of the 19 WorldClim bioclimatic variables (BIO1–BIO17) computed at 415 georeferenced occurrence localities of *Agrobacterium tumefaciens* using JMP Pro 16. Upper triangular panels display Pearson correlation coefficients (r); circle size is proportional to −log_10_(p-value) reflecting statistical significance; colour indicates direction and magnitude of correlation (deep red = strong positive, r → +1.0; deep blue = strong negative, r → −1.0; pale grey = negligible correlation). Lower triangular panels show bivariate scatter plots with 95% confidence ellipses. Diagonal cells identify each variable by name. Variables with |r| ≥ 0.80 were removed as collinear; the five retained predictors — BIO1, BIO2, BIO3, BIO14, and BIO15 — show comparatively lower intercorrelation values.

### 3.2 Model evaluation and validation

The MaxEnt model demonstrated excellent predictive performance across all 10 bootstrap replicates. The mean AUC was 0.959 (± narrow standard deviation as illustrated by the tight blue band around the mean ROC curve), far exceeding the random expectation of 0.5 (**Figure 2**). The TSS reached a value of 0.77, well above the 0.70 threshold widely regarded as indicative of excellent model performance, confirming that the model’s simultaneous capacity to correctly predict presences and absences was not attributable to chance (Fielding and Bell, 1997; Allouche et al., 2006). The BIOCLIM envelope test further corroborated these findings, placing 364 of 404 evaluated observations (90.1%) within the defined climatic envelope at the training site level, while 307 records (76%) fell within the envelope when evaluated globally, indicating a robust and ecologically coherent niche characterisation (**Figure 2**).

**Figure 2:**
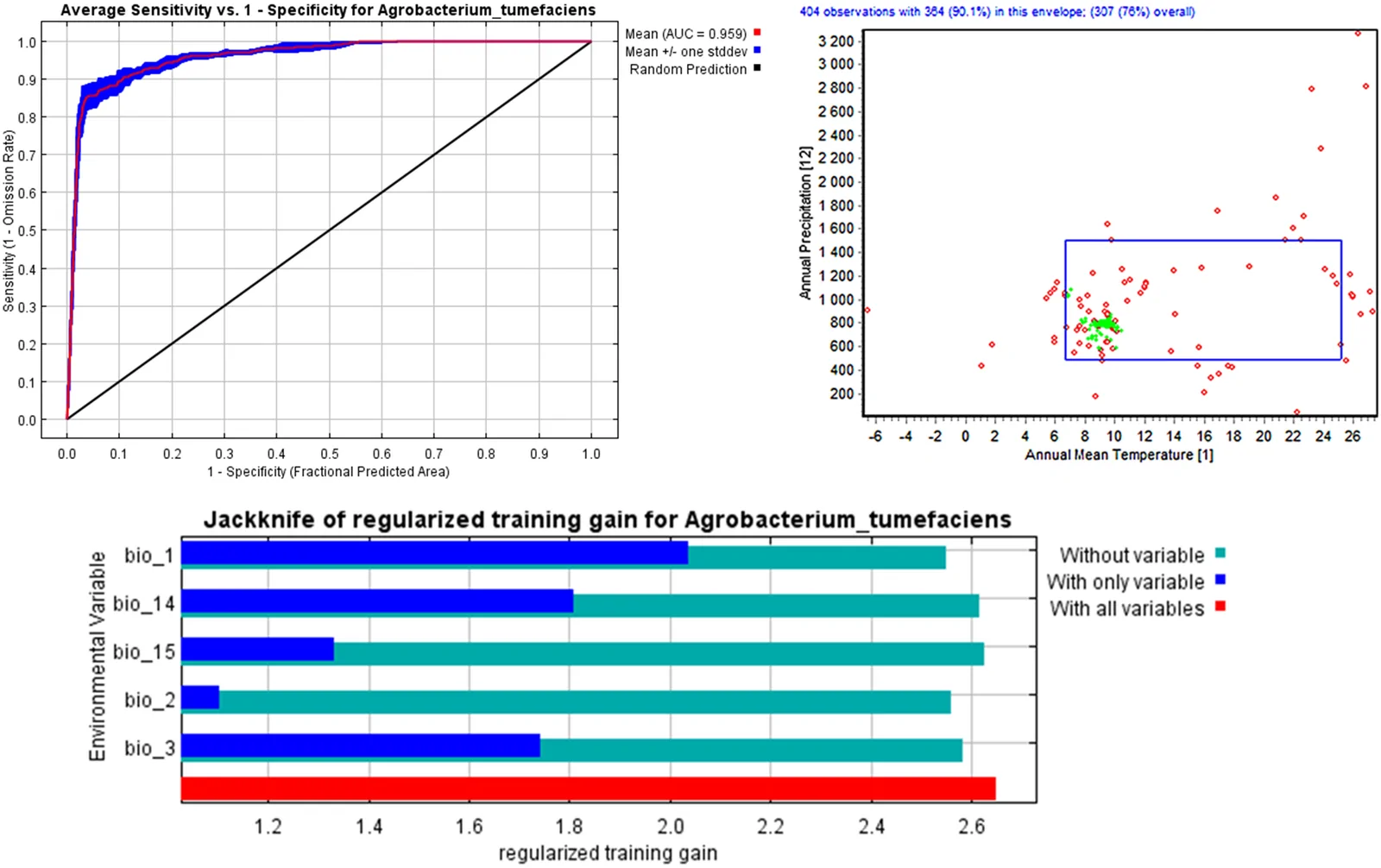
Model evaluation and variable importance for *Agrobacterium tumefaciens*. (Top left) Mean ROC curve averaged across 10 bootstrap replicates; red line = mean AUC (0.959); blue band = ± 1 SD; diagonal black line = random prediction (AUC = 0.5). (Top right) BIOCLIM envelope test in BIO1 (annual mean temperature, x-axis) vs. BIO12 (annual precipitation, y-axis) climatic space; blue rectangle = bioclimatic training envelope; green diamonds = occurrence points inside the envelope (364/404; 90.1%); red diamonds = outside the envelope; 307 records (76%) fell within the envelope globally. (Bottom) Jackknife test of regularised training gain; teal bars = model gain when each variable is excluded; dark blue bars = gain when each variable is used alone; red bar = full model gain using all five variables.

### 3.3 Bioclimatic variable importance: jackknife analysis and response curves

The jackknife test of regularised training gain confirmed that all five retained bioclimatic variables contributed meaningfully and independently to model performance, though with clearly differentiated levels of importance (**Figure 2**). BIO1 (mean annual temperature) emerged as the single most informative variable when used in isolation, achieving the highest individual training gain among all predictors, while its omission from the full model produced the greatest reduction in gain — collectively establishing BIO1 as the primary climatic driver structuring the distribution of *A. tumefaciens* at the global scale. BIO14 (precipitation of the driest month) ranked second in terms of unique explanatory power, followed by BIO15 (precipitation seasonality), BIO3 (isothermality), and BIO2 (mean diurnal temperature range). The response curves further illuminated the ecological relationships underlying these variable contributions (**Figure 3**). The BIO1 curve revealed a threshold-type response, with predicted suitability rising sharply and peaking at mean annual temperatures between approximately 8 °C and 20 °C, declining steeply at higher values and remaining near zero in the coldest thermal regimes. The BIO2 response showed a strong monotonic decline from high suitability at low diurnal ranges (around 40–60 units) toward near-zero suitability above 120 units. BIO3 displayed a unimodal response peaking near isothermality values of 25–35. BIO14 showed peak suitability at low but non-zero driest-month precipitation values (approximately 10–50 mm), declining sharply under complete seasonal drought. BIO15 exhibited a markedly negative relationship with precipitation seasonality, with the highest suitability concentrated in regions where rainfall is distributed relatively evenly throughout the year.

**Figure 3:**
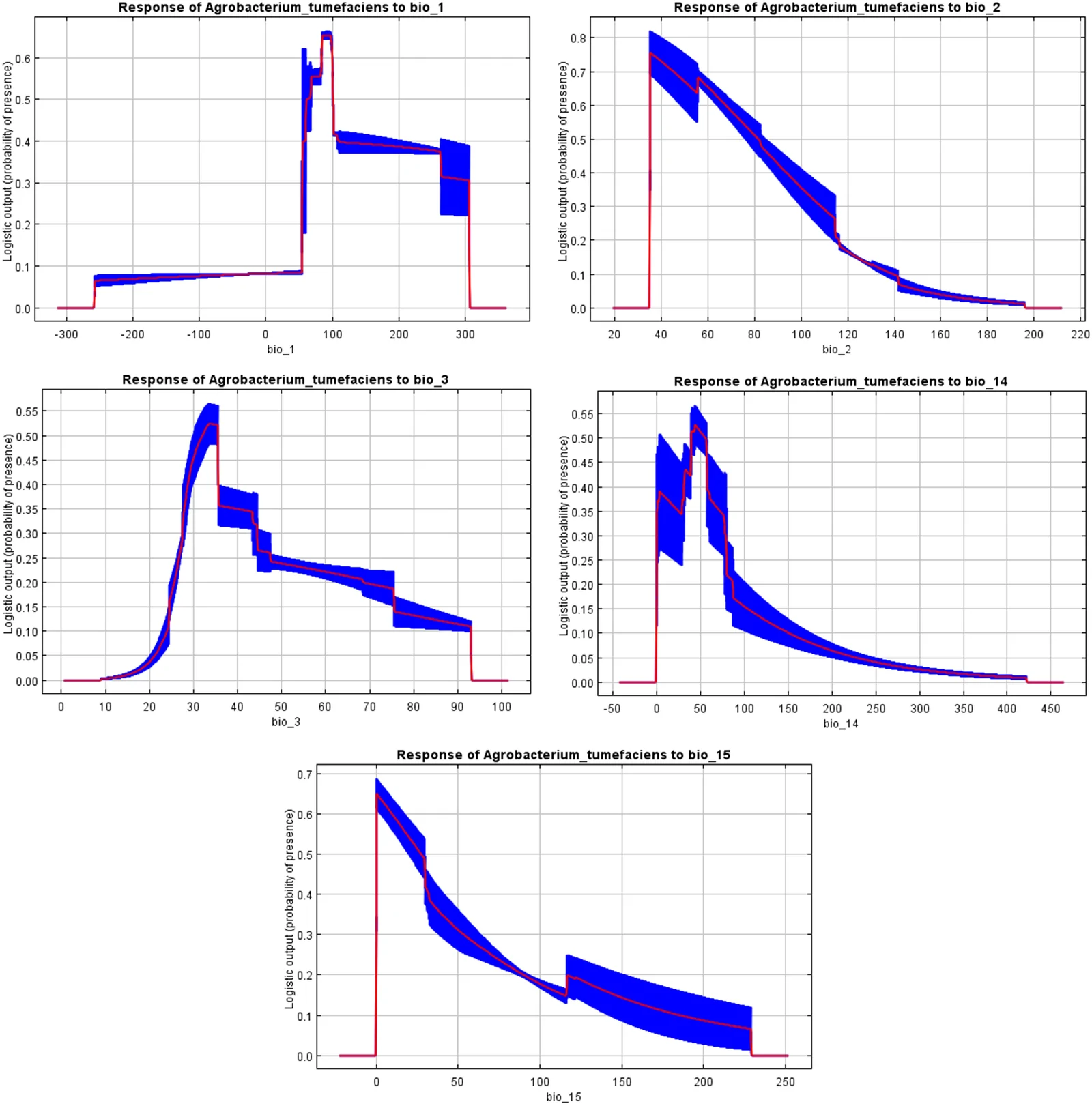
MaxEnt response curves for *Agrobacterium tumefaciens* illustrating the relationship between predicted habitat suitability (logistic output; y-axis) and each of the five selected bioclimatic variables (x-axis). Red lines represent the mean response across 10 bootstrap replicates; blue shading denotes ±1 SD. Variables shown: BIO1 (mean annual temperature, ×0.1 °C), BIO2 (mean diurnal temperature range, ×0.1 °C), BIO3 (isothermality, dimensionless), BIO14 (precipitation of the driest month, mm), and BIO15 (precipitation seasonality, coefficient of variation). All other variables were held at their mean values during curve generation.

### 3.4 Current predicted distribution of *Agrobacterium tumefaciens*

The MaxEnt model projected a geographically structured and climatically coherent pattern of habitat suitability for *Agrobacterium tumefaciens* under current baseline climatic conditions, with suitable areas distributed across temperate and warm-temperate zones of both hemispheres while the vast majority of the global terrestrial surface — encompassing boreal forests, arctic tundra, tropical rainforests, and hyper-arid deserts — was classified as unsuitable (**Figure 4**). In the Northern Hemisphere, the most concentrated zones of high and very high suitability were identified across western and central Europe, where conditions in the British Isles, France, Germany, the Balkans, northern Italy, and extending into Ukraine and the western margins of Russia supported the highest predicted probability of presence. This European core emerged as the globally most suitable region under current climate, reflecting the combination of moderate mean annual temperatures, low diurnal temperature ranges, intermediate isothermality values, and relatively even year-round precipitation distribution. In North America, a well-defined band of medium to high suitability extended across the eastern United States and the Great Lakes region, while the arid interior plains, the Rockies, and Canada’s boreal zone remained largely unsuitable. Across Asia, notable corridors of low to medium suitability were projected along the foothills of the Himalayas, across parts of northern India, and extending into southern and eastern China, the Korean Peninsula, and Japan.

**Figure 4:**
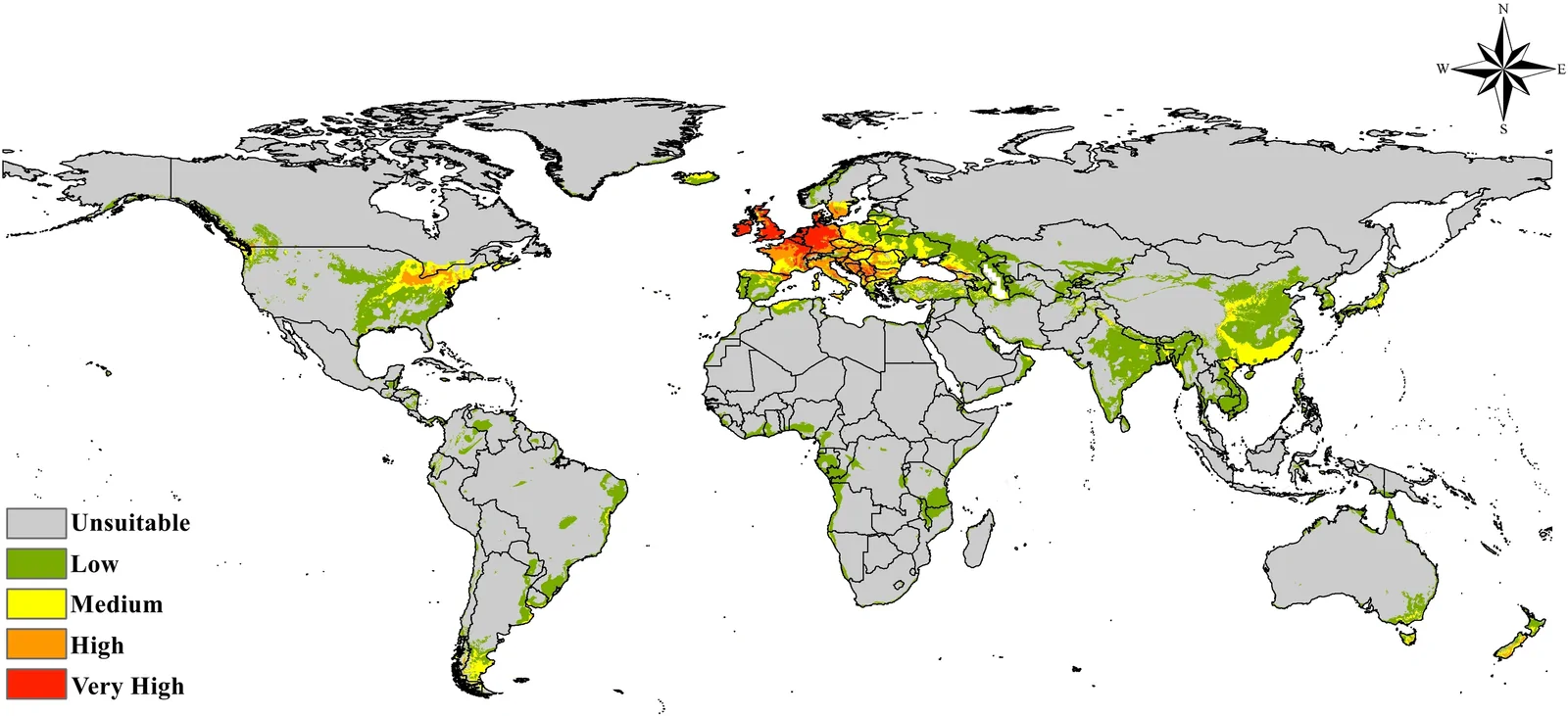
Predicted current global distribution of habitat suitability for *Agrobacterium tumefaciens* generated by MaxEnt under WorldClim 2.1 baseline climate (reference period 1970–2000). Suitability is classified as: Unsuitable (grey, 0–0.20), Low (green, 0.20–0.40), Medium (yellow, 0.40–0.60), High (orange, 0.60–0.80), and Very High (red, 0.80–1.00). Map produced in ArcGIS 10.8 using the WGS 1984 geographic coordinate system.

In the Southern Hemisphere, suitable habitat was considerably more fragmented and geographically restricted, yet identifiable clusters of low to medium suitability were nonetheless projected across southern Brazil, the temperate coastal zones of Chile and Argentina, isolated portions of eastern and southern Africa including highland areas of Ethiopia, Kenya, and South Africa, and small pockets along the southwestern and southeastern coasts of Australia and in New Zealand. Tropical equatorial regions were almost entirely projected as unsuitable, while cold high-latitude and high-altitude environments were excluded by thermal minima falling below the bacterium’s lower survival threshold. These results establish a well-defined global baseline distribution against which future climate-driven range shifts can be systematically assessed. It should be noted that this current suitability surface reflects the pathogen’s realised climatic niche as inferred from documented occurrence records and therefore corresponds closely to regions where crown gall disease has already been reported; by contrast, the future projections presented in Section 3.5 identify additional climatically permissive areas beyond the currently documented range, which should be interpreted as zones of emerging risk rather than confirmed presence.

### 3.5 Future predicted distribution under climate change scenarios

The MaxEnt projections under the four future climate scenarios revealed a broadly consistent trend of northward and altitudinal range shifts in the potential distribution of *Agrobacterium tumefaciens*, though the magnitude and geographic pattern of these changes varied substantially between the two emission pathways and across the two time horizons (**Figure 5**). Under the moderate mitigation scenario RCP 2.6 in 2050 (**Figure 5a**), the overall spatial footprint of suitable habitat remained broadly comparable to the current baseline, with the core European zone of high and very high suitability largely maintained while notable expansions of medium suitability were projected across eastern Europe, Central Asia, and an enlarged corridor through northern India and eastern China. Under RCP 8.5 in 2050 (**Figure 5b**), the redistribution of suitability became more pronounced, with medium and low suitability classes expanding more extensively into higher latitudes across Scandinavia, Russia’s western margins, and northern China, while the highest suitability classes began a perceptible contraction within the formerly core European and eastern American zones. The 2070 projections amplified these trends considerably. Under RCP 2.6 in 2070 (**Figure 5c**), suitability patterns showed moderate continued reorganisation relative to 2050 with low suitability areas maintaining a broad geographic presence. The most dramatic reconfiguration was projected under RCP 8.5 in 2070 (**Figure 5d**), where extensive areas of formerly high and very high suitability showed clear degradation, while simultaneously new areas of low to medium suitability emerged at higher latitudes — a pattern consistent with a thermally driven poleward and upslope displacement of the species’ climatic niche.

**Figure 5:**
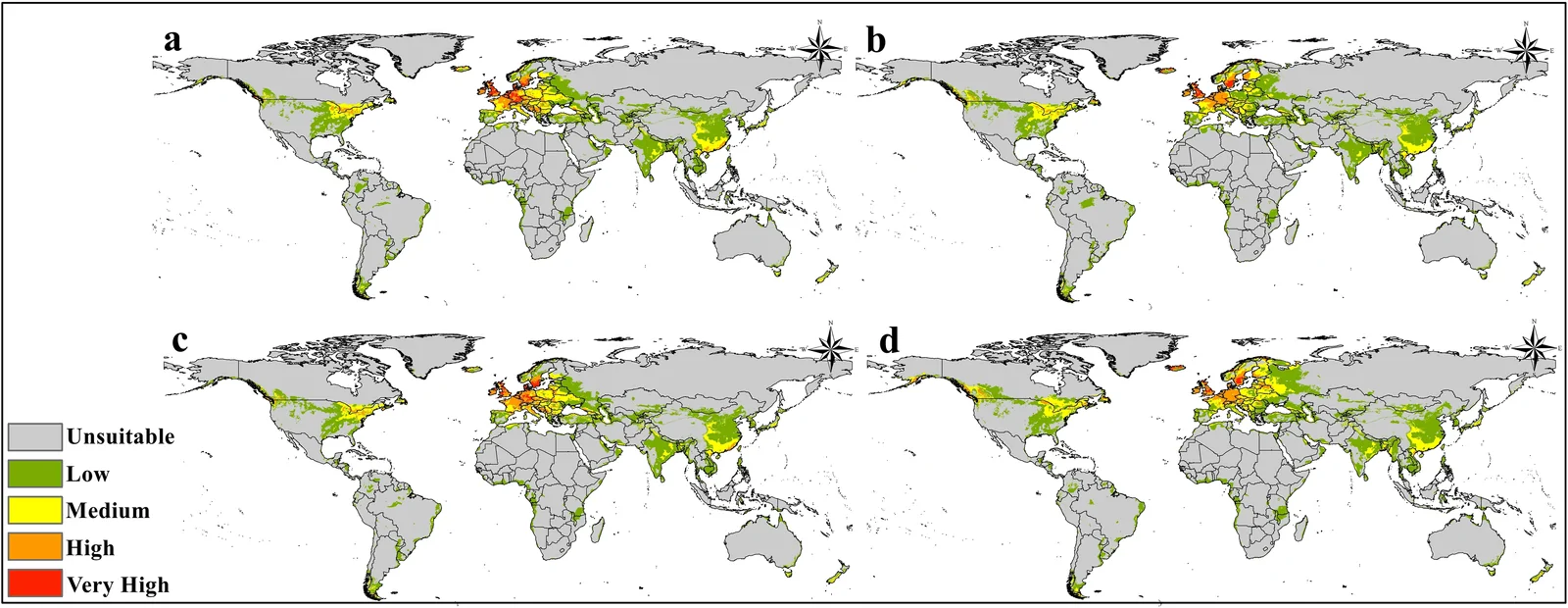
Projected future potential distribution of habitat suitability for *Agrobacterium tumefaciens* under four climate change scenarios using the BCC-CSM1–1 general circulation model: **(a)** 2050 RCP 2.6; **(b)** 2050 RCP 8.5; **(c)** 2070 RCP 2.6; **(d)** 2070 RCP 8.5. Suitability classification as in **Figure 4**. Maps produced in ArcGIS 10.8; WGS 1984 coordinate system.

### 3.6 Projected changes in suitable habitat area across scenarios

The change detection maps derived by overlaying current and future binary suitability surfaces revealed a spatially heterogeneous mosaic of habitat gain, loss, and stability across all four scenarios (**Figure 6**). Habitat loss was concentrated primarily within the climatically warmest portions of the current range — notably the Mediterranean basin, the southeastern United States, lowland South Asia, and parts of East Asia. Habitat gain was most extensive under the RCP 8.5 scenarios, particularly by 2070, expanding into previously unsuitable higher-latitude regions of northern Europe, central Russia, northern China, and parts of Canada. Stable suitable areas persisted most consistently across temperate Europe and the northeastern United States, underscoring these regions as climatic refugia likely to support *A. tumefaciens* populations throughout the century regardless of emission trajectory. The quantitative summary of these spatial changes is presented in **Table 1**.

**Figure 6:**
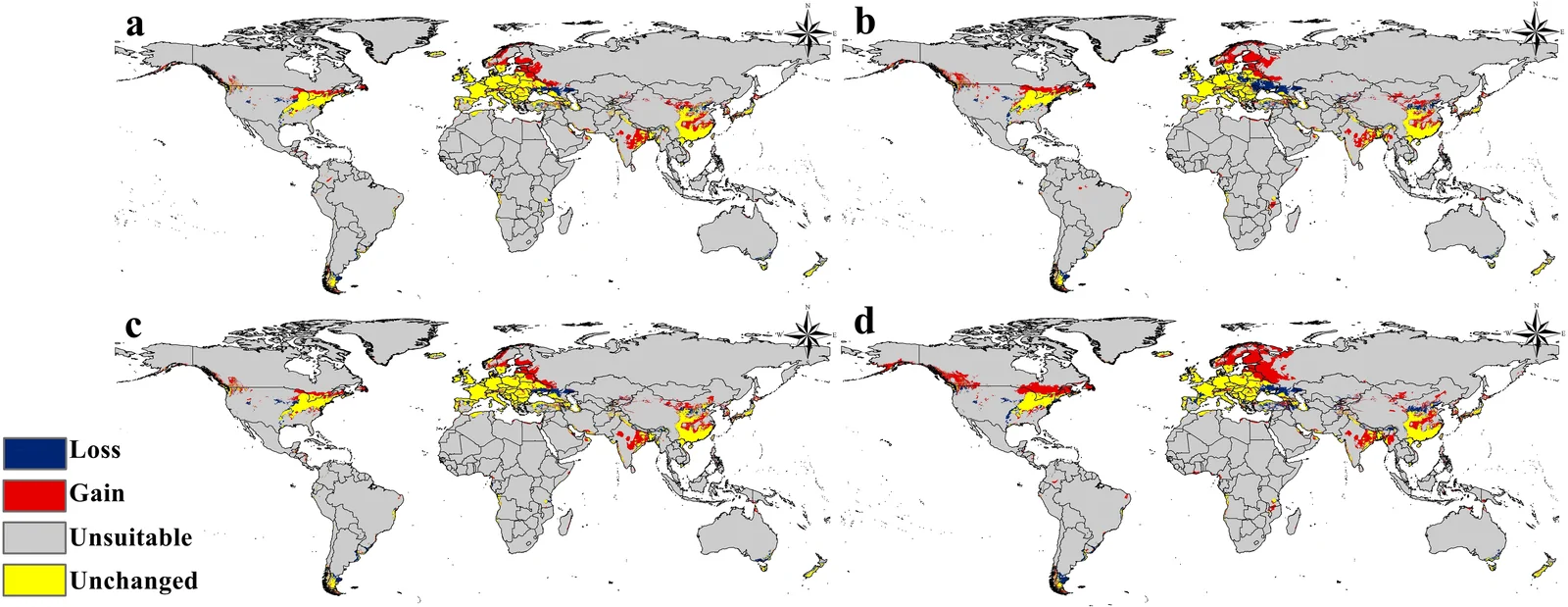
Change detection maps showing spatial gains, losses, and stable suitable habitat for *Agrobacterium tumefaciens* relative to the current baseline under four future climate scenarios: **(a)** 2050 RCP 2.6; **(b)** 2050 RCP 8.5; **(c)** 2070 RCP 2.6; **(d)** 2070 RCP 8.5. Red = newly gained suitable habitat; dark blue = lost suitable habitat; yellow = stable suitable habitat retained from baseline; grey = persistently unsuitable.

**Table 1:** Projected changes in global terrestrial habitat suitability area (%) for *Agrobacterium tumefaciens* across four future climate scenarios relative to the current baseline.

Suitability class	Current (%)	2050 RCP 2.6 (%)	2050 RCP 8.5 (%)	2070 RCP 2.6 (%)	2070 RCP 8.5 (%)
Very High (0.80–1.00)	2.1	1.8	1.5	1.6	0.9
High (0.60–0.80)	3.4	3.1	2.7	2.9	1.8
Medium (0.40–0.60)	5.8	6.2	6.7	6.0	5.4
Low (0.20–0.40)	9.3	10.1	11.4	10.6	12.7
Unsuitable (0–0.20)	79.4	78.8	77.7	78.9	79.2
Net Suitable Area	20.6	21.2	22.3	21.1	20.8
Habitat Gain (vs. current)	—	+3.1	+5.8	+2.9	+4.7
Habitat Loss (vs. current)	—	−2.5	−4.1	−2.4	−6.3
Net Change (vs. current)	—	+0.6	+1.7	+0.5	−1.5

Values represent percentage of global terrestrial area in each suitability class. Net change = gain minus loss relative to current baseline.

### 3.7 Limiting factor analysis

The limiting factor map generated through the BIOCLIM envelope analysis in DIVA-GIS revealed a spatially differentiated pattern of climatic constraints governing the potential distribution of *Agrobacterium tumefaciens* across the global terrestrial surface (**Figure 7**). Mean annual temperature (BIO1, light green) emerged as the principal limiting factor across the broadest spatial extent, dominating across high-latitude regions of North America — particularly the central and northern United States interior — as well as across large portions of northern and central Asia, confirming that thermal insufficiency is the most widespread constraint preventing the establishment of *A. tumefaciens* in currently cold-limited regions. Mean diurnal temperature range (BIO2, medium green) acted as the dominant limiting variable across the semi-arid and continental interior zones of North America, central Asia, the Arabian Peninsula, and parts of the African Sahel, regions where extreme day-to-night temperature fluctuations impose chronic physiological stress on bacterial cell membranes. Isothermality (BIO3, teal) was identified as the primary limiting factor across a geographically coherent belt spanning tropical and subtropical Africa, the Indian subcontinent, parts of Southeast Asia, and northern South America. Precipitation of the driest month (BIO14, medium blue) dominated across the arid and semi-arid zones of sub-Saharan Africa, the Middle East, southwestern Asia, and the drier interior regions of Australia and South America. Precipitation seasonality (BIO15, dark navy blue) emerged as the primary constraint across parts of eastern China, southeastern Asia, the Horn of Africa, and coastal regions of South America. Taken together, the limiting factor map underscores that the global distribution of this pathogen is governed by a spatially variable interplay of thermal and hydrological limitations rather than a single universal constraint.

**Figure 7:**
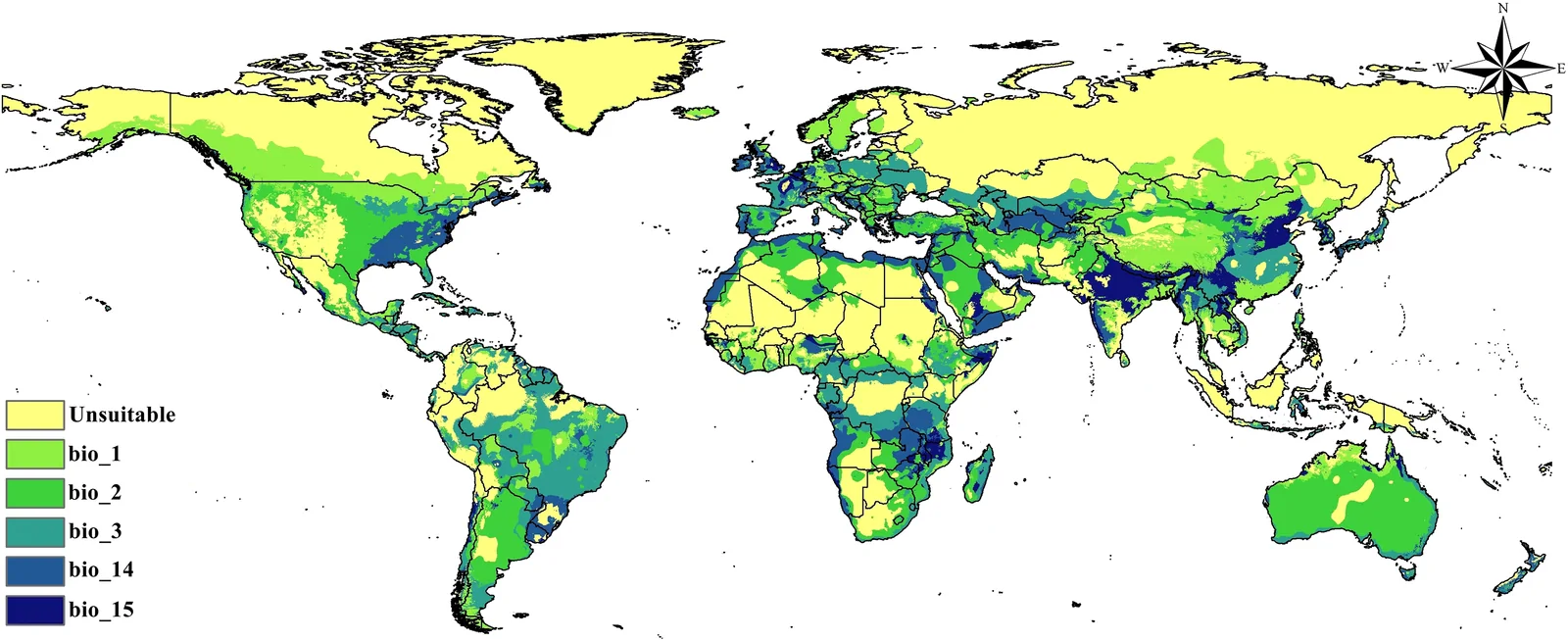
Global limiting factor map for *Agrobacterium tumefaciens* generated using the BIOCLIM envelope model in DIVA-GIS 7.5. Each grid cell is coloured by the single bioclimatic variable most strongly limiting suitability at that location relative to training occurrence conditions. Colour categories: Unsuitable (yellow); BIO1 — mean annual temperature (light green); BIO2 — mean diurnal temperature range (medium green); BIO3 — isothermality (teal); BIO14 — precipitation of the driest month (medium blue); BIO15 — precipitation seasonality (dark navy blue). Map produced in ArcGIS 10.8; WGS 1984 coordinate system.

## 4 Discussion

The present study provides, to the best of our knowledge, one of relatively few comprehensive global assessments of the climatic niche and potential distribution of *Agrobacterium tumefaciens* under current and future climate conditions, using the MaxEnt framework calibrated on 415 georeferenced occurrence records and five carefully selected non-collinear bioclimatic predictors. The model’s excellent validation performance — AUC = 0.959 and TSS = 0.77 — indicates a high degree of confidence in the spatial predictions generated (Fielding and Bell, 1997; Allouche et al., 2006). We emphasise, however, that MaxEnt is a correlative algorithm that characterises statistical associations between occurrence and environment rather than causal physiological mechanisms (Elith and Leathwick, 2009); the ecological interpretations offered below are therefore best understood as climatically informed hypotheses consistent with known aspects of the pathogen’s biology, rather than as direct mechanistic proof. AUC values reported for SDMs of other soil-borne plant pathogens, including *Ralstonia solanacearum* (Faye et al., 2020), *Fusarium oxysporum* (Alkhalifah et al., 2023), and *Phytophthora cinnamomi* (Burgess et al., 2017), fall in a broadly similar range to that obtained here; because AUC is not directly comparable across studies that differ in species, occurrence datasets, background extent, and modelling choices (see Discussion, Limitations), we treat this correspondence as contextual rather than as formal evidence of superior performance, and place greater weight below on whether the predicted spatial pattern itself is consistent with the documented geography of other bacterial plant pathogens. The narrow standard deviation bands observed around the mean ROC curve across 10 bootstrap replicates additionally indicate that the model was stable and not overly sensitive to the particular random subsample of occurrence records used in any individual run, a desirable property given the inherent stochasticity of background point selection in MaxEnt (Merow et al., 2013).

The identification of mean annual temperature (BIO1) as the single most important predictor of *A. tumefaciens* distribution is ecologically well-grounded and corroborates the known physiological characteristics of this mesophilic bacterium. *Agrobacterium tumefaciens* exhibits optimal growth rates between approximately 22 °C and 28 °C, with virulence gene expression on the Ti plasmid being particularly temperature-sensitive: vir gene induction is strongly inhibited above 30 °C and sharply reduced below 14 °C (Fullner and Nester, 1996). The threshold-type BIO1 response curve identified in the present study, with suitability rising sharply between 8 °C and 20 °C and declining steeply at higher values, is therefore climatically coherent and consistent with the known biokinetic temperature range documented for related mesophilic soil bacteria (Alqahtani et al., 2025); we note that direct physiological confirmation for *A. tumefaciens* specifically would require laboratory validation beyond the scope of this correlative modelling study. Similar thermal constraints have been identified as the primary drivers of distribution in other soil bacterial pathogens modelled with SDM approaches, including *Streptomyces scabies* (Khatabi et al., 2021) and *Xanthomonas campestris* (Singh et al., 2022), suggesting that thermal niche conservatism may be a general feature of phytopathogenic prokaryotes that warrants systematic investigation in future macroecological studies.

The strong negative relationship between predicted suitability and mean diurnal temperature range (BIO2) reflects the physiological intolerance of *A. tumefaciens* to large within-day thermal fluctuations, which impose repeated stress on bacterial cell membrane integrity and disrupt the stable rhizosphere microenvironment required for chemotactic movement toward wounded root surfaces and subsequent infection (Tecon and Or, 2017). This finding aligns with the broader principle that diurnal temperature variability is an important but often under-appreciated axis of the climatic niche for soil microorganisms, as noted in global analyses of bacterial biogeography (Fierer et al., 2009). The unimodal response to isothermality (BIO3) further reinforces the interpretation that *A. tumefaciens* occupies a climatic space defined by thermal stability rather than extremes — a characteristic shared by many temperate-adapted soil microbiota that rely on consistent seasonal cycles for population dynamics and sporadic infection windows coinciding with plant wounding during agricultural operations (Dechesne et al., 2010).

The importance of precipitation seasonality (BIO15) and driest-month precipitation (BIO14) as determinants of distribution highlights the critical role of soil moisture dynamics in governing bacterial survival, dispersal, and pathogenicity. Soil water potential directly controls the connectivity of water films through which *A. tumefaciens* navigates the soil pore network toward susceptible root tissue (Or et al., 2007), and severe dry-season deficits have been shown to dramatically reduce bacterial population densities in agricultural soils even when temperature conditions remain within the optimal range (Or et al., 2007; Tecon and Or, 2017). The strongly negative suitability response to high BIO15 values indicates that regions with pronounced seasonal alternation between wet and dry conditions — such as Mediterranean-climate zones, monsoon-influenced tropical systems, and semi-arid continental interiors — are intrinsically less suitable for *A. tumefaciens*. This has important practical implications: areas with high agricultural vulnerability to crown gall that currently experience moderate BIO15 values may cross critical thresholds of precipitation variability under climate change, potentially causing paradoxical suitability losses despite general warming (Araujo and Luoto, 2007). From an applied perspective, these projections suggest two concrete priorities for phytosanitary planning: first, nursery and rootstock certification programmes in regions currently at the margin of climatic suitability — for example, northern France, southern England, the Balkans, and parts of the Pacific Northwest of North America — merit closer surveillance, as these areas are projected to shift toward medium suitability under RCP 8.5 (**Table 1**; **Figure 6**); second, plant-health authorities in currently core high-suitability regions projected to lose suitability, such as the Mediterranean basin and the southeastern United States, may wish to progressively reallocate long-term monitoring resources toward the higher-latitude zones identified here as emerging risk areas, rather than assuming continuity of historical disease pressure in traditionally affected production regions.

The current distribution model identified western and central Europe as the globally most suitable region for *A. tumefaciens*, a prediction entirely consistent with the long-documented history of crown gall as a major constraint on viticulture, stone fruit production, and ornamental horticulture in this region (Burr and Otten, 1999; Pulawska, 2010). The strong secondary zones of suitability projected across the eastern United States, northern India, and eastern China similarly mirror the geographic distribution of reported crown gall outbreaks in these major agricultural regions (Savary et al., 2019). The reasonable correspondence between modelled suitability and known disease distribution provides additional ecological validation of the model beyond the quantitative AUC and TSS metrics and supports the interpretation that the five retained bioclimatic variables adequately capture the principal axes of the pathogen’s fundamental climatic niche.

The future projections collectively indicate that climate change will not produce a simple uniform expansion or contraction of *A. tumefaciens* distribution, but rather a complex spatial reorganisation characterised by simultaneous gains at higher latitudes and losses within portions of the current core range — a pattern consistent with the range shift dynamics documented for numerous other organisms under projected warming (Thuiller et al., 2008; Warren et al., 2018). The progressive poleward displacement of suitable habitat is most marked under RCP 8.5 by 2070, where northern European countries, parts of Scandinavia, the Baltic states, and higher-elevation zones of Central Asia appear for the first time as climatically permissive for the pathogen. This finding carries substantial practical significance because these regions host extensive areas of previously disease-naive viticulture and fruit tree cultivation, where growers, extension services, and regulatory authorities have historically had little reason to implement crown gall management protocols (Savory et al., 2022). The emergence of climatic suitability in these novel areas, if realised through pathogen dispersal via infected planting material — the primary vehicle of long-distance *A. tumefaciens* movement globally (Burr et al., 1998) — could represent a significant and largely unprepared biosecurity challenge in the coming decades.

Conversely, the projected degradation of very high suitability zones in the Mediterranean basin, the southeastern United States, lowland South Asia, and parts of East Asia under the high-emission scenarios reflects the increasing thermal stress and precipitation seasonality that warming is expected to impose on these currently core regions. Several of these areas — particularly the Mediterranean basin — are simultaneously projected to experience intensified summer drought under future climate scenarios (Giorgi and Lionello, 2008), compounding the effect of thermal exceedance on BIO15 and BIO14 constraints identified by the limiting factor analysis. The limiting factor map produced through the BIOCLIM envelope analysis in DIVA-GIS provides complementary, spatially explicit insight into the climatic constraints governing *A. tumefaciens* distribution that is difficult to infer from MaxEnt suitability surfaces alone. By identifying the single variable most responsible for excluding the pathogen from each unsuitable grid cell, the map offers actionable intelligence for disease risk communication: regions where BIO1 is the primary barrier are those most likely to become newly suitable as warming progresses, while regions dominated by BIO14 or BIO15 constraints are those where precipitation-management interventions in irrigated systems might create local pockets of suitability even without further climate change (Vrain, 1987).

Several limitations of the present study merit acknowledgment. The MaxEnt model, as a correlative SDM, assumes that the species is in equilibrium with its current climate and does not explicitly model dispersal barriers, biotic interactions, evolutionary adaptation, or edaphic properties that are known to modulate *A. tumefaciens* virulence and persistence (Araujo et al., 2019). Because several of the edaphic variables known to modulate *A. tumefaciens* persistence and virulence (soil pH, texture, organic matter content) often correlate with the bioclimatic predictors used here (Fick and Hijmans, 2017; Karger et al., 2017), we expect the current model to capture some of their indirect signal, but direct incorporation of high-resolution soil data (e.g., ISRIC SoilGrids) would allow this assumption to be tested explicitly in future work. The MaxEnt model was also run using default regularization and feature-class settings; while these defaults have been shown to perform robustly for broadly distributed, well-sampled species (Phillips and Dudik, 2008; Merow et al., 2013), formal tuning of model complexity using established frameworks (Warren and Seifert, 2011; Radosavljevic and Anderson, 2014) would allow the bias–variance trade-off to be optimised more rigorously and is identified here as a priority for the next iteration of this work.

The reliance on default regularization and a single GCM is common practice in the applied MaxEnt literature: a recent systematic review of species distribution modelling studies published between 2015 and 2019 found that the majority (65%) relied on a single model configuration rather than a tuned ensemble (Bahram et al., 2018; Santini et al., 2021), and several previous MaxEnt-based assessments of soil and plant-associated microorganisms using the same modelling pipeline and a single GCM under RCP 2.6 and RCP 8.5 including *Staphylococcus aureus* (Alqahtani et al., 2025), *Pseudomonas syringae* (Khalaf et al., 2024a), and *Aspergillus niger* (Alkhalifah et al., 2022) have been peer-reviewed and published using this approach. This does not make the practice methodologically ideal, but it indicates that our design choices are consistent with, rather than exceptional relative to, the current state of the field. On the specific question of model tuning, we expect its effect on our conclusions to be modest rather than qualitative: the dominant predictor (BIO1) shows a strong, ecologically coherent threshold-type response, the model achieves high and stable discrimination (AUC = 0.959; TSS = 0.77) with narrow variance across 10 bootstrap replicates, and default-versus-tuned MaxEnt comparisons in the wider literature typically alter the smoothness of response curves and the extent of fine-scale suitability boundaries more than they alter which variables dominate or the overall direction of predicted range shifts (Warren and Seifert, 2011; Radosavljevic and Anderson, 2014). We therefore expect that formal tuning would refine, rather than reverse, the patterns reported here, but we cannot rule out shifts in the precise area estimates in **Table 1** and flag this as a genuine source of quantitative, though probably not qualitative, uncertainty. On the choice of a single GCM, the empirical evidence on GCM-related uncertainty in SDM projections indicates that between-GCM variability in projected suitability is generally smaller than between-emission-scenario variability in the near term, but grows to a comparable or larger magnitude by the end of the century, particularly under high-emission trajectories (Goberville et al., 2015). This matters directly for our results: it suggests that the qualitative direction of the projected changes a poleward displacement of suitable habitat and contraction of very high suitability in the current core range, a pattern that recurs across the broader climate–pest and climate–pathogen literature independent of specific GCM choice (Bebber et al., 2013; Garrett et al., 2006; Thuiller et al., 2008) is likely to be comparatively robust, whereas the precise magnitude of habitat gain and loss we report for 2070 under RCP 8.5 (**Table 1**) is the result most likely to shift under a multi-GCM ensemble, and should accordingly be read as illustrative of the scale of change rather than as a precise quantitative forecast.

Additionally, while the 415 occurrence records retained after filtering represent a substantial dataset for a bacterial pathogen with inherently sparse distributional data, geographic sampling bias with Africa, South America, and Australasia comparatively underrepresented — may cause the model to underestimate suitability in data-poor regions. This limitation should be addressed in future studies through targeted field surveys and culture-independent soil metagenomic analyses capable of detecting *A. tumefaciens* presence without reliance on classical isolation methods. Notwithstanding these limitations, the results of this study represent a meaningful and timely advance in our understanding of the macroecological dynamics of one of the world’s most important plant bacterial pathogens under a rapidly changing climate.

## 5 Conclusion

This study presents, to our knowledge, one of the first global MaxEnt-based assessments of the climatic niche and potential distribution of *Agrobacterium tumefaciens* under current and future climate scenarios, contributing: (i) a spatially explicit global baseline against which future surveillance can be benchmarked; (ii) a scenario-based projection framework spanning two emission pathways and two time horizons that quantifies both habitat gain and loss rather than net change alone; and (iii) a limiting factor analysis that disaggregates the climatic drivers of unsuitability by geographic region, information not recoverable from suitability maps alone. The analytical workflow applied here is not specific to this pathogen and could be extended to other soil-borne bacterial or fungal plant pathogens for which occurrence and climate data are available. The model, validated with AUC = 0.959 and TSS = 0.77, identified mean annual temperature as the dominant climatic driver, with precipitation of the driest month, precipitation seasonality, isothermality, and mean diurnal temperature range acting as complementary limiting factors across different geographic regions. Future projections under RCP 2.6 and RCP 8.5 for 2050 and 2070 consistently indicated a poleward displacement of suitable habitat, with progressive gains at higher latitudes and losses in parts of the current core range, the severity of which scales with emission trajectory and time horizon. These findings have concrete implications for plant health surveillance, biosecurity policy, and the design of climate-adaptive crown gall management strategies, and underscore the urgent need to incorporate climate change projections into phytosanitary risk frameworks for soil-borne bacterial pathogens.

## Data Availability

All data supporting the findings of this study are included within the article and its **Supplementary Material**, including the full list of occurrence records used for model calibration (**Supplementary Table S1**). Further inquiries can be directed to the corresponding author.
