# Supplementary material for: Climate change and the potential global distribution of Agrobacterium tumefaciens: a MaxEnt modeling approach for current and future climate scenarios
Source: Front Plant Sci. 2026 Sep 25;17:1941149. doi: 10.3389/fpls.2026.1941149 (PMC13649741; doi:10.3389/fpls.2026.1941149)
Supplement: Supplementary file 1 [file Table1.pdf]

Sp.	Latitude	Longitude
Agrobacterium tumefa(	52.76536	-0.87904
Agrobacterium tumefa(	32.0544	78.1933
Agrobacterium tumefa(	49.25581	-122.712
Agrobacterium tumefa(	49.25037	-122.619
Agrobacterium tumefa(	50.61387	-118.739
Agrobacterium tumefa(	43.55981	-83.5342
Agrobacterium tumefa(	43.42069	-82.8038
Agrobacterium tumefa(	25.5941	85.1376
Agrobacterium tumefa(	35.37803	-77.5605
Agrobacterium tumefa(	43.86475	-83.3046
Agrobacterium tumefa(	40.66	-73.95
Agrobacterium tumefa(	22.32	107.48
Agrobacterium tumefa(	40.7	-73.82
Agrobacterium tumefa(	40.66	-73.98
Agrobacterium tumefa(	40.68	-73.87
Agrobacterium tumefa(	51.8	5.85
Agrobacterium tumefa(	51.8	5.8
Agrobacterium tumefa(	52.3	4.85
Agrobacterium tumefa(	53.1	6.4
Agrobacterium tumefa(	51.4	5.45
Agrobacterium tumefa(	51.8	4.65
Agrobacterium tumefa(	51.85	4.15
Agrobacterium tumefa(	51.95	5.9
Agrobacterium tumefa(	51.5	6.1
Agrobacterium tumefa(	52.05	6.1
Agrobacterium tumefa(	52.25	6.1
Agrobacterium tumefa(	52.15	5.4
Agrobacterium tumefa(	51.75	4.75
Agrobacterium tumefa(	52.25	5.25
Agrobacterium tumefa(	50.85	5.9
Agrobacterium tumefa(	53.45	5.7
Agrobacterium tumefa(	51.95	5.65
Agrobacterium tumefa(	51.9	4.55
Agrobacterium tumefa(	53.35	6.2
Agrobacterium tumefa(	52.3	5.6
Agrobacterium tumefa(	51.95	4.35
Agrobacterium tumefa(	52.35	4.6
Agrobacterium tumefa(	51.7	4.7
Agrobacterium tumefa(	52.05	5.1
Agrobacterium tumefa(	53.15	4.85
Agrobacterium tumefa(	53.15	6.55
Agrobacterium tumefa(	51.8	4.5
Agrobacterium tumefa(	51.6	5.4

Agrobacterium tumefa	52.3	4.5
Agrobacterium tumefa	51.9	4.5
Agrobacterium tumefa	51.75	5.5
Agrobacterium tumefa	52.3	5.8
Agrobacterium tumefa	52.1	5
Agrobacterium tumefa	52.2	5.6
Agrobacterium tumefa	52	4.35
Agrobacterium tumefa	51.9	6
Agrobacterium tumefa	51.85	6
Agrobacterium tumefa	52.8	6.85
Agrobacterium tumefa	51.95	6.25
Agrobacterium tumefa	51.65	5.25
Agrobacterium tumefa	52.1	5.35
Agrobacterium tumefa	52.3	5.55
Agrobacterium tumefa	51.15	5.85
Agrobacterium tumefa	51.75	4.65
Agrobacterium tumefa	51.2	5.95
Agrobacterium tumefa	51.8	4.75
Agrobacterium tumefa	52.55	4.6
Agrobacterium tumefa	39.20025	-84.4242
Agrobacterium tumefa	52	6
Agrobacterium tumefa	52.15	6.7
Agrobacterium tumefa	52.35	5.25
Agrobacterium tumefa	53.25	6.35
Agrobacterium tumefa	52.1	6.7
Agrobacterium tumefa	52.65	5.85
Agrobacterium tumefa	53	6.55
Agrobacterium tumefa	52.1	6.65
Agrobacterium tumefa	52.1	5.1
Agrobacterium tumefa	51.05772	3.73485
Agrobacterium tumefa	50.15085	5.09267
Agrobacterium tumefa	51.20101	4.66753
Agrobacterium tumefa	50.22613	4.89967
Agrobacterium tumefa	51.07621	3.6495
Agrobacterium tumefa	50.79969	4.41195
Agrobacterium tumefa	51.95	4.3
Agrobacterium tumefa	51.8	4.8
Agrobacterium tumefa	52.6	6
Agrobacterium tumefa	51.8	5.4
Agrobacterium tumefa	51.7	4.75
Agrobacterium tumefa	51.85	5.5
Agrobacterium tumefa	52	4.3
Agrobacterium tumefa	52.15	5.35
Agrobacterium tumefa	53.88633	10.75622

Agrobacterium tumefa(	52.1	5.05
Agrobacterium tumefa(	51.4	5.8
Agrobacterium tumefa(	52.35	6.1
Agrobacterium tumefa(	51.9	4.45
Agrobacterium tumefa(	51.55	5.45
Agrobacterium tumefa(	51.7	4.6
Agrobacterium tumefa(	51.3	6.05
Agrobacterium tumefa(	51.7	5.8
Agrobacterium tumefa(	51.8	4.3
Agrobacterium tumefa(	51.9	5.95
Agrobacterium tumefa(	51.95	5.95
Agrobacterium tumefa(	51.4	4.35
Agrobacterium tumefa(	51.95	4.5
Agrobacterium tumefa(	52.35	6.15
Agrobacterium tumefa(	51.25	3.95
Agrobacterium tumefa(	51.75	4.8
Agrobacterium tumefa(	52.5	6.05
Agrobacterium tumefa(	52.5	6.35
Agrobacterium tumefa(	52.55	5.8
Agrobacterium tumefa(	51.7	4.8
Agrobacterium tumefa(	52.5	6
Agrobacterium tumefa(	51.6	4.65
Agrobacterium tumefa(	51.9	4.7
Agrobacterium tumefa(	53.15	6
Agrobacterium tumefa(	53.15	6.2
Agrobacterium tumefa(	52.35	5.1
Agrobacterium tumefa(	52.55	6.6
Agrobacterium tumefa(	53.15	6.4
Agrobacterium tumefa(	52.85	6
Agrobacterium tumefa(	51.75	5.7
Agrobacterium tumefa(	51.85	4.65
Agrobacterium tumefa(	51.7	5.85
Agrobacterium tumefa(	47.59332	10.26519
Agrobacterium tumefa(	51.42947	7.030824
Agrobacterium tumefa(	53.98903	10.82722
Agrobacterium tumefa(	53.90442	10.72128
Agrobacterium tumefa(	52.01233	9.882894
Agrobacterium tumefa(	47.54702	10.28771
Agrobacterium tumefa(	51.33089	8.264422
Agrobacterium tumefa(	52.10001	9.762073
Agrobacterium tumefa(	51.99217	9.865925
Agrobacterium tumefa(	48.8636	9.288983
Agrobacterium tumefa(	47.56451	10.23544
Agrobacterium tumefa(	52.31591	9.851

Agrobacterium tumefa	51.81405	11.78906
Agrobacterium tumefa	53.70507	9.501577
Agrobacterium tumefa	47.56257	10.71287
Agrobacterium tumefa	47.38371	11.27961
Agrobacterium tumefa	52.45392	13.40401
Agrobacterium tumefa	51.8485	12.42142
Agrobacterium tumefa	48.89312	9.43342
Agrobacterium tumefa	50.62757	8.533802
Agrobacterium tumefa	47.51772	10.28063
Agrobacterium tumefa	48.29389	10.09306
Agrobacterium tumefa	47.56587	10.25145
Agrobacterium tumefa	47.55568	10.08974
Agrobacterium tumefa	47.45189	10.28003
Agrobacterium tumefa	48.84808	9.402452
Agrobacterium tumefa	47.64469	10.30719
Agrobacterium tumefa	48.72816	9.092649
Agrobacterium tumefa	52.51746	13.34704
Agrobacterium tumefa	52.55016	10.14982
Agrobacterium tumefa	52.31768	9.555571
Agrobacterium tumefa	50.82376	8.790651
Agrobacterium tumefa	49.01794	8.403406
Agrobacterium tumefa	49.94963	8.657255
Agrobacterium tumefa	47.51923	10.2596
Agrobacterium tumefa	47.6138	10.2602
Agrobacterium tumefa	49.94446	8.45788
Agrobacterium tumefa	48.72395	9.086723
Agrobacterium tumefa	51.81143	10.34569
Agrobacterium tumefa	50.56491	8.388748
Agrobacterium tumefa	53.85287	10.62948
Agrobacterium tumefa	52.52359	13.28994
Agrobacterium tumefa	53.83476	11.89915
Agrobacterium tumefa	53.10531	8.239145
Agrobacterium tumefa	54.31059	10.78952
Agrobacterium tumefa	47.48694	10.28269
Agrobacterium tumefa	47.47464	10.30389
Agrobacterium tumefa	47.69546	10.03965
Agrobacterium tumefa	51.98567	9.887867
Agrobacterium tumefa	51.7	5.55
Agrobacterium tumefa	52.65	4.75
Agrobacterium tumefa	52.7	6.25
Agrobacterium tumefa	52.3	6.55
Agrobacterium tumefa	52.05	4.2
Agrobacterium tumefa	51.85	4.55
Agrobacterium tumefa	51.8	5

Agrobacterium tumefac	52.8	4.65
Agrobacterium tumefac	52	5.5
Agrobacterium tumefac	52.65	6.6
Agrobacterium tumefac	51.85	5.9
Agrobacterium tumefac	52.15	4.45
Agrobacterium tumefac	51.8	4.35
Agrobacterium tumefac	52.3	5.25
Agrobacterium tumefac	52.75	6.2
Agrobacterium tumefac	52.75	6.35
Agrobacterium tumefac	51.65	5.4
Agrobacterium tumefac	51.8	5.75
Agrobacterium tumefac	51.85	6.05
Agrobacterium tumefac	51.8	6
Agrobacterium tumefac	51.85	4.85
Agrobacterium tumefac	52.45	4.6
Agrobacterium tumefac	51.7	3.8
Agrobacterium tumefac	52.5	4.95
Agrobacterium tumefac	52.5	5.95
Agrobacterium tumefac	52.4	6.5
Agrobacterium tumefac	50.8	5.65
Agrobacterium tumefac	52.5	5.05
Agrobacterium tumefac	51.9	4.2
Agrobacterium tumefac	51.8	4.6
Agrobacterium tumefac	51.95	5.1
Agrobacterium tumefac	53.25	6.1
Agrobacterium tumefac	51.95	4.9
Agrobacterium tumefac	51.9	5.65
Agrobacterium tumefac	52.3	5.1
Agrobacterium tumefac	51.75	5.9
Agrobacterium tumefac	53.2	6.3
Agrobacterium tumefac	51.9	4.6
Agrobacterium tumefac	51.95	5.5
Agrobacterium tumefac	51.75	4.7
Agrobacterium tumefac	51.95	4.45
Agrobacterium tumefac	51.3	5.45
Agrobacterium tumefac	51.85	5.6
Agrobacterium tumefac	51.9	4.8
Agrobacterium tumefac	52.6	6.5
Agrobacterium tumefac	53.45	5.65
Agrobacterium tumefac	52.25	6.5
Agrobacterium tumefac	50.85	5.65
Agrobacterium tumefac	51.6	5.45
Agrobacterium tumefac	51.55	6.05
Agrobacterium tumefac	52.05	4.45

Agrobacterium tumefac	52	5.65
Agrobacterium tumefac	51.95	4.95
Agrobacterium tumefac	50.85	5.85
Agrobacterium tumefac	51.95	5.3
Agrobacterium tumefac	51.65	5.2
Agrobacterium tumefac	53.3	6.65
Agrobacterium tumefac	51.9	4.35
Agrobacterium tumefac	51.95	4.25
Agrobacterium tumefac	51.95	5.7
Agrobacterium tumefac	51.75	5.75
Agrobacterium tumefac	53.2	6.1
Agrobacterium tumefac	52.25	4.95
Agrobacterium tumefac	52.25	6.75
Agrobacterium tumefac	51.9	5.25
Agrobacterium tumefac	51.6	5.5
Agrobacterium tumefac	50.75	5.85
Agrobacterium tumefac	52.15	5.95
Agrobacterium tumefac	52.4	4.6
Agrobacterium tumefac	52.15	4.5
Agrobacterium tumefac	52.4	6.45
Agrobacterium tumefac	52.1	4.45
Agrobacterium tumefac	52.15	5.3
Agrobacterium tumefac	52.7	6.4
Agrobacterium tumefac	52.65	5
Agrobacterium tumefac	51.8	4.4
Agrobacterium tumefac	51.9	5.15
Agrobacterium tumefac	52.7	4.65
Agrobacterium tumefac	51.75	5.85
Agrobacterium tumefac	52.3	5.75
Agrobacterium tumefac	52	4.2
Agrobacterium tumefac	50.9	5.85
Agrobacterium tumefac	52.65	4.7
Agrobacterium tumefac	51.5	5
Agrobacterium tumefac	51.95	5.45
Agrobacterium tumefac	51.9	6.2
Agrobacterium tumefac	52.4	6.1
Agrobacterium tumefac	52.35	6.65
Agrobacterium tumefac	52.45	4.8
Agrobacterium tumefac	53.05	4.85
Agrobacterium tumefac	50.85	5.7
Agrobacterium tumefac	51.95	6.7
Agrobacterium tumefac	51.45	6.05
Agrobacterium tumefac	53.2	5.95
Agrobacterium tumefac	51.9	6.1

Agrobacterium tumefa	52.7	5.2
Agrobacterium tumefa	51.95	6.75
Agrobacterium tumefa	51.85	5.95
Agrobacterium tumefa	52.1	4.95
Agrobacterium tumefa	51.45	5.95
Agrobacterium tumefa	51.85	4.5
Agrobacterium tumefa	52.3	4.6
Agrobacterium tumefa	52.25	4.55
Agrobacterium tumefa	51.85	5.4
Agrobacterium tumefa	53.15	6.15
Agrobacterium tumefa	52	4.15
Agrobacterium tumefa	52.15	4.75
Agrobacterium tumefa	52.35	5.6
Agrobacterium tumefa	52.05	4.35
Agrobacterium tumefa	52.6	5.95
Agrobacterium tumefa	50.85	6.05
Agrobacterium tumefa	53.3	6.1
Agrobacterium tumefa	52.55	5.9
Agrobacterium tumefa	52	6.4
Agrobacterium tumefa	51.35	5.1
Agrobacterium tumefa	52.35	5.55
Agrobacterium tumefa	51.8	5.9
Agrobacterium tumefa	51.6	5.1
Agrobacterium tumefa	52.05	4.25
Agrobacterium tumefa	52.4	6.8
Agrobacterium tumefa	52.4	6.65
Agrobacterium tumefa	52.2	5.05
Agrobacterium tumefa	51.6	5.15
Agrobacterium tumefa	52.05	5.25
Agrobacterium tumefa	52.3	5.9
Agrobacterium tumefa	52.1	4.65
Agrobacterium tumefa	53.15	5.75
Agrobacterium tumefa	52.55	6.55
Agrobacterium tumefa	52.4	6.4
Agrobacterium tumefa	51.45	5.45
Agrobacterium tumefa	52.65	6.25
Agrobacterium tumefa	52.2	6.75
Agrobacterium tumefa	51.65	5.1
Agrobacterium tumefa	51.6	5
Agrobacterium tumefa	51.5	4.45
Agrobacterium tumefa	52.3	4.8
Agrobacterium tumefa	52	5.9
Agrobacterium tumefa	52.35	5.8
Agrobacterium tumefa	51.95	4.65

Agrobacterium tumefa	52.15	6.9
Agrobacterium tumefa	51.9	4.25
Agrobacterium tumefa	52.1	6.15
Agrobacterium tumefa	52.1	5.3
Agrobacterium tumefa	52.1	5.25
Agrobacterium tumefa	51.65	5.95
Agrobacterium tumefa	52.9	6.1
Agrobacterium tumefa	52.2	6.25
Agrobacterium tumefa	51.45	4.3
Agrobacterium tumefa	51.7	4.3
Agrobacterium tumefa	52.5	4.65
Agrobacterium tumefa	51.45	3.85
Agrobacterium tumefa	51.55	5.95
Agrobacterium tumefa	51.65	3.7
Agrobacterium tumefa	52.3	6.5
Agrobacterium tumefa	51.8	5.6
Agrobacterium tumefa	52	4.55
Agrobacterium tumefa	52.2	4.9
Agrobacterium tumefa	52.05	5.4
Agrobacterium tumefa	52.35	6.05
Agrobacterium tumefa	51.95	5.8
Agrobacterium tumefa	51.4	3.85
Agrobacterium tumefa	52	6.05
Agrobacterium tumefa	51.5	4.85
Agrobacterium tumefa	52.7	6.95
Agrobacterium tumefa	52.35	4.9
Agrobacterium tumefa	52.1	5.55
Agrobacterium tumefa	52.35	5.75
Agrobacterium tumefa	52	4.7
Agrobacterium tumefa	51.55	5
Agrobacterium tumefa	51.8	3.9
Agrobacterium tumefa	52.35	5.15
Agrobacterium tumefa	52.1	4.4
Agrobacterium tumefa	51.25	3.4
Agrobacterium tumefa	52.15	6.2
Agrobacterium tumefa	52.65	4.6
Agrobacterium tumefa	51.4	6.1
Agrobacterium tumefa	52.95	5.85
Agrobacterium tumefa	51.5	5.25
Agrobacterium tumefa	52.25	4.75
Agrobacterium tumefa	53.4	5.75
Agrobacterium tumefa	52.05	4.85
Agrobacterium tumefa	50.8	5.85
Agrobacterium tumefa	51.8	5.65

Agrobacterium tumefa	51.45	6.1
Agrobacterium tumefa	52	5.7
Agrobacterium tumefa	51.9	5.9
Agrobacterium tumefa	52.65	6.2
Agrobacterium tumefa	51.8	4.9
Agrobacterium tumefa	52.9	5.9
Agrobacterium tumefa	50.95	5.85
Agrobacterium tumefa	23.5863	81.173
Agrobacterium tumefa	38.07325	104.6911
Agrobacterium tumefa	-1.10307	-78.5547
Agrobacterium tumefa	-1.98534	-79.0769
Agrobacterium tumefa	7.480134	80.61903
Agrobacterium tumefa	6.90649	80.92137
Agrobacterium tumefa	6.141744	81.11938
Agrobacterium tumefa	53.3	6.25
Agrobacterium tumefa	51.35	3.4
Agrobacterium tumefa	18.52	73.99
Agrobacterium tumefa	28.11	81.59
Agrobacterium tumefa	27.62	85.58
Agrobacterium tumefa	36.23	71.29
Agrobacterium tumefa	36.08	68.42
Agrobacterium tumefa	37.35	69.51
Agrobacterium tumefa	43.15478	-77.6156
Agrobacterium tumefa	47.60621	-122.332
Agrobacterium tumefa	42.43983	-76.4953
Agrobacterium tumefa	40.88928	-72.6466
Agrobacterium tumefa	42.44063	-76.4966
Agrobacterium tumefa	40.81677	-73.0662
Agrobacterium tumefa	43.02979	-76.0044
Agrobacterium tumefa	42.51396	-76.3466
Agrobacterium tumefa	46.71621	-122.954
Agrobacterium tumefa	42.45182	-76.4812
Agrobacterium tumefa	25.6	85.12
Agrobacterium tumefa	-16.5766	-39.558
Agrobacterium tumefa	52.27509	-3.33783
Agrobacterium tumefa	3.27199	-73.0877
Agrobacterium tumefa	54.09	28.23
Agrobacterium tumefa	53.92	27.69
Agrobacterium tumefa	41.46278	-8.2675
Agrobacterium tumefa	21.5	105.44
Agrobacterium tumefa	34.4	105.06
Agrobacterium tumefa	37.52	102.5
Agrobacterium tumefa	34.05	105.41
Agrobacterium tumefa	31.47	-84.29

Agrobacterium tumefac	22.59	5.4
Agrobacterium tumefac	21.76	78.87
Agrobacterium tumefac	12.29	93.85
Agrobacterium tumefac	35.42	4.24
Agrobacterium tumefac	10.38	124.56
Agrobacterium tumefac	42.0927	-86.3563
Agrobacterium tumefac	22.57	72.88
Agrobacterium tumefac	37.98	23.91
Agrobacterium tumefac	-16.26	-49.24
Agrobacterium tumefac	-18.94	-46.99
Agrobacterium tumefac	18.16	74.5
Agrobacterium tumefac	25.11	85.33
Agrobacterium tumefac	37.62	-120.76
Agrobacterium tumefac	27.47	94.91
Agrobacterium tumefac	-29.8191	25.3499
Agrobacterium tumefac	19.45	-90.09
Agrobacterium tumefac	47.58	21.46
Agrobacterium tumefac	36.69	10.5
Agrobacterium tumefac	36.18	8.71
